# A Non-Biological Method for Screening Active Components against Influenza Virus from Traditional Chinese Medicine by Coupling a LC Column with Oseltamivir Molecularly Imprinted Polymers

**DOI:** 10.1371/journal.pone.0084458

**Published:** 2013-12-26

**Authors:** Ya-Jun Yang, Jian-Yong Li, Xi-Wang Liu, Ji-Yu Zhang, Yu-Rong Liu, Bing Li

**Affiliations:** Gansu Provincial Engineering Research Center for New Animal Drug, Key Laboratory of New Animal Drug Project, Key Laboratory of Veterinary Pharmaceutical Development, Ministry of Agriculture, Lanzhou Institute of Husbandry and Pharmaceutical Sciences of CAAS, Lanzhou, Gansu Province, China; Kliniken der Stadt Köln gGmbH, Germany

## Abstract

To develop a non-biological method for screening active components against influenza virus from traditional Chinese medicine (TCM) extraction, a liquid chromatography (LC) column prepared with oseltamivir molecularly imprinted polymer (OSMIP) was employed with LC-mass spectrometry (LC-MS). From chloroform extracts of compound TCM liquid preparation, we observed an affinitive component *m/z* 249, which was identified to be matrine following analysis of phytochemical literatures, OSMIP-LC column on-line of control compounds and MS/MS off-line. The results showed that matrine had similar bioactivities with OS against avian influenza virus H9N2 *in vitro* for both alleviating cytopathic effect and hemagglutination inhibition and that the stereostructures of these two compounds are similar while their two-dimensional structures were different. In addition, our results suggested that the bioactivities of those affinitive compounds were correlated with their chromatographic behaviors, in which less difference of the chromatographic behaviors might have more similar bioactivities. This indicates that matrine is a potential candidate drug to prevent or cure influenza for human or animal. In conclusion, the present study showed that molecularly imprinted polymers can be used as a non-biological method for screening active components against influenza virus from TCM.

## Introduction

Molecular imprinting has been recognized as a technique for the ready preparation of polymers containing recognition sites of predetermined specificity. Molecularly imprinted polymers (MIPs) are called “plastic antibodies” with substrate affinities comparable to those of antibodies. MIPs have, therefore, been developed for a variety of applications in enantiomer separation [1,2], solid-phase extraction [3,4], analytical chemistry [5,6], chemical and biomimetic sensors [7–9], and drug delivery [10–12], etc. The perfect selectivity, high binding affinity and physical robustness of MIPs enable them to be used for non-biological screening in drug discovery [9,13–15]. The structures of affinitive components, which were trapped by MIPs from matrix, had similarity to the template. These affinitive components would have the similar bioactivity to the template from a structure-activity relationship (SAR) point of view. MIPs have now been used for screening analogues with similar bioactivities to search for new drug candidates from plant extracts [16,17] or combinatorial chemistry libraries [18]. 

**Figure 1:**
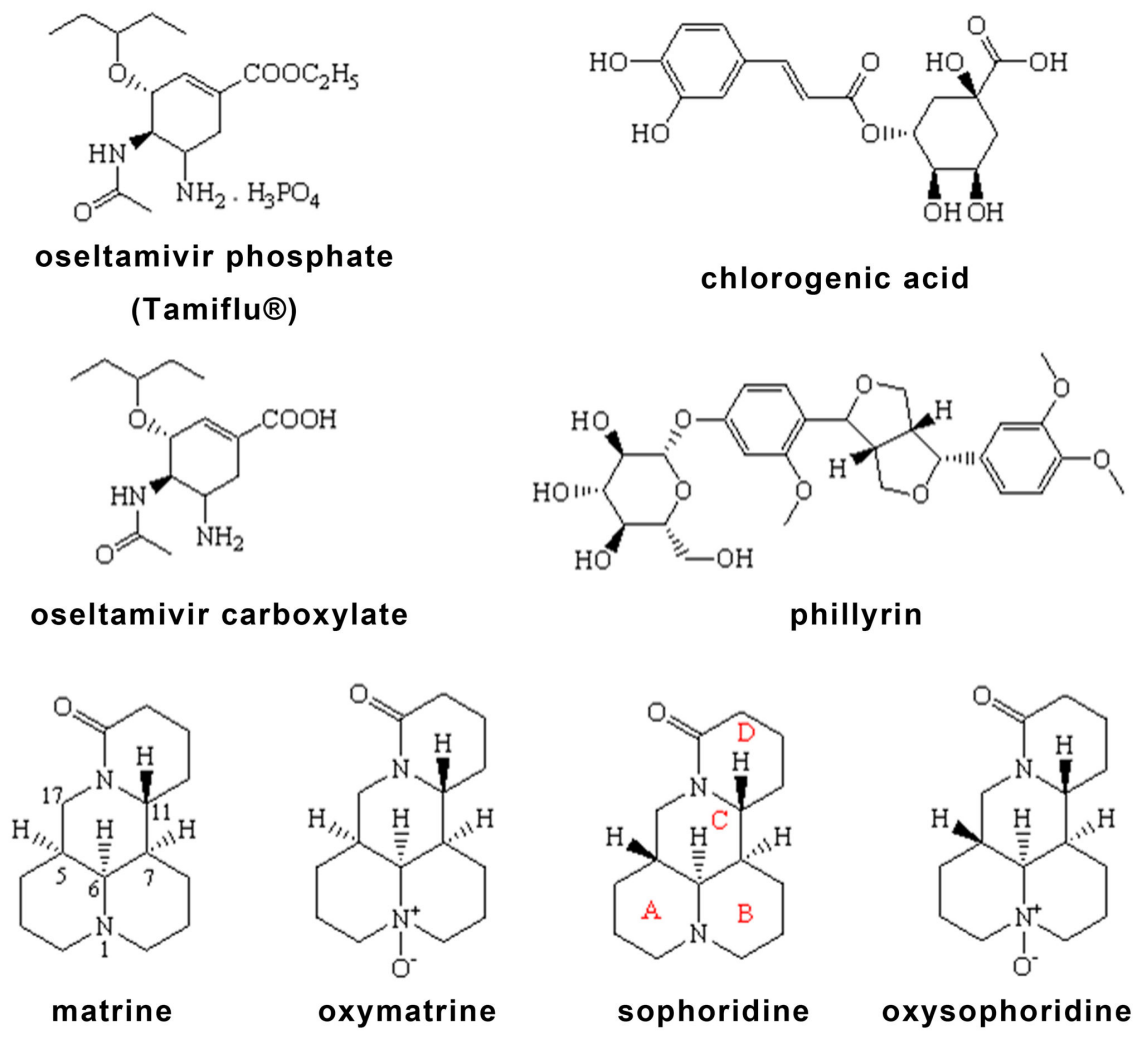
Chemical structures of OS and other compounds.

Pandemic influenza is caused by a naturally occurring pathogen and is generally considered as the most significant potential global public health emergency. Recently, human cases of highly pathogenic strains of avian influenza (H5N1) have raised the concerns of the imminence of this threat. On June 11th, 2009, the World Health Organization signaled that a global pandemic of novel influenza A (H1N1) was underway by raising the worldwide pandemic alert level to Phase 6. The important role of wild birds has been proved in introduction and spread of H5N1 subtype in different countries in Asia and other continents [19–21]. A large number of chickens have been killed and buried deeply when chickens or other poultries infected with highly pathogenic strains of avian influenza virus (H5N1 or other subtypes) were found in those regions. Vaccination was tried to protect human and animals from new subtypes of influenza virus. Oseltamivir (OS; see Figure 1), the ethyl ester prodrug of the neuraminidase inhibitor oseltamivir carboxylate, has been licensed for the treatment of patients with influenza virus infection. OS is considered the leading antivirus currently available to control outbreak of influenza [22]. On the other hand, it is necessary to develop new drugs against the virus including pandemic influenza, avian influenza H5N1 and other subtypes. Furthermore, there are increasing literatures reporting the development of oseltamivir-resistant of influenza virus [23–26]. However, highly pathogenic strains of avian influenza virus is dangeous to humans and traditional screening for antivirus drug is a tedious and higher dangerous work with strict requirements for laboratory conditions. In order to accelerate the screening processes for antivirus drug development, it is important to develop a feasibly biological replacement method. 

Herbs have been applied to protect people from common colds and influenza for thousands of years. Traditional Chinese Medicine (TCM) is different from modern medicine. The mechanisms of TCM is very complicated, including expelling pathogens through sweating, urination, strengthening the hosts’ immune system, making the them less susceptible to the pathogens, including influenza, etc. In China, many physicians believe that herbs are effective against colds and influenza. Some Chinese herbs have been demonstrated to be antiviral, antiasthmatic, antitussive and antipyretic. Herbs for various symptoms or causes are combined in different quantities as a basic prescription to treat colds and influenza in different seasons. Some active compounds against influenza virus have been separated and identified from traditional Chinese herbs (TCH) which were used for colds or influenza therapy. For example, *Scutellariae Radix* has antiphlogistic effect [27] and *Glycyrrhizae Radix et Rhizoma* has expectorant and antitussive effects [28]. *Sophorae Flavescentis Radix* combined with other TCH is used in colds and influenza treatment. Some pterocarpans and flavanones have been isolated from *Sophora flavescens* which was shown to inhibit neuraminidase. Pterocarpan 1 is the best inhibitor with an IC_50_ of 1.4 µM [29].

In our previous work [30], OS-MIPs were synthesized and packed into stainless steel liquid chromatography (LC) column. The specific affinity tests of OSMIP for the template molecule OS and other different compounds such as chlorogenic acid, phillyrin, aspirin and diisooctyl phthalate, have been investigated. The results showed that OSMIP had a very high affinity and selectivity to separate the template molecule from other compounds. Therefore, this column with OSMIP was used to search for more active OS analogs from TCH extraction in the present study. We found that some compounds from TCH possessed high affinity with the column, among which one compound could inhibit influenza virus *in vitro*. We also reported here for the first time that the compound with different two-dimensional structures from the template molecule OS shared its antiviral effect.

## Materials and Methods

### Analytic equipment

The analytic systems consisted of an Agilent 1200 HPLC with a MassHunter software version B.01.04 (Agilent Technologies, USA) and an Agilent 6410A triple-quadrupole (QQQ) mass spectrometer (Agilent Technologies, USA) with an ESI source interface operated in the positive-ion scan mode.

###  Chemicals and preparation

4-Vinylpyridine (4-VP) was bought from Alfa Aesar (Lancashire, England). Acrylamide (AA), ethylene glycol dimethacrylate (EGDMA) and azobisisobutyronitrile (AIBN) were purchased from Shanghai No. 4 Reagent and H.V. Chemical Co., Ltd. (Shanghai, China). Before use, EGDMA was distilled under vacuum after being extracted with 10% sodium hydroxide brine and dried over anhydrous magnesium sulfate. AIBN was recrystallized from alcohol. 4-VP was extracted with 10% sodium hydroxide brine and dried over anhydrous magnesium sulfate. Toluene was dried with sodium and then redistilled. 

Methanol (MeOH) and acetonitrile were HPLC grade (Fisher Scientific, New Jersey, USA). MeOH, chloroform, ethyl acetate, petroleum ether (60 - 90 °C) and formic acid were analytical-reagent grade (Sinopharm Chemical Reagent Co, Ltd). Deionized water (18 MΩ) was prepared with a Direct-Q®3 system (Millipore, USA).

Oseltamivir phosphate (Roche R&D Center (China) Ltd.) was neutralized and extracted using sodium hydroxide and ethyl acetate, and then the extraction was vacuum dried at 50 °C. Chlorogenic acid, phillyrin, matrine, oxymatrine, sophoridine, oxysophoridine and aspirin were purchased from National Institute for Food and Drug Control (Beijing, China). TCH containing *Lonicerae Japonicae Flos*, *Isatidis Radix*, *Sophorae Flavescentis Radix*, *Forsythiae Fructus*, *Commelinae* Herba and others were bought from licensed Good Supplying Practice (GSP) drugstore (Lanzhou, China). Compound liquid preparation of TCM “*Yandureqing*” containing *Lonicerae Japonicae Flos*, *Isatidis Radix*, *Sophorae Flavescentis Radix*, *Forsythiae Fructus* and *Commelinae Herba* was prepared by water extracting - alcohol precipitating in our lab as described previously [31], and one milliliter of this liquid preparation was equal to 2 grams dried herbs mixture.

### Preparation of MIPs

OS-MIP was synthesized under the optimized condition as described previously [30]. Briefly, 0.1 mmol OS (template) and 0.69 mmol 30% AA + 70% 4-VP (functional monomer) were dissolved in 5 ml of toluene (porogen) in a 100-ml flask and the flask was placed in an ultrasonic water bath for 30 min, and then shaken for 3 h. After 5.0 mmol EGDMA (cross-linker) and 15 mg AIBN (initiator) were added to the solution, the mixture was purged with nitrogen for 5 min with the flask under an airtight seal. The polymerization proceeded in a water bath at 60 °C for 24 h. The block polymer obtained was ground using a laboratory mortar and pestle and particles between 25 and 45 μm were collected after the smaller particles were removed via repeated sedimentation in acetone. The polymer particles were wet-packed into stainless steel columns (150 mm × 4.6 mm i.d.) with MeOH under pressure of 2 000 psi. The columns were sequentially washed with MeOH, MeOH-acetic acid 9:1 (*v/v*) and MeOH until a stable baseline indicating removal of the template molecules. 

### Sample Preparation

The extractions with different polarity were obtained from these herbs’ extractum by common method. They were dried in vacuum at 50 °C and redissolved with MeOH. One milliliter of this MeOH solution was equal to 10 grams dried herb. Extractions from TCM liquid preparation were carried out using direct extracting with different polarity solvents in turn following the procedures stated above. One milliliter of this MeOH solution was equal to 5 milliliter liquid preparation. These MeOH solutions were passed through 0.22 μm organic filter films for the following affinitive screening with OSMIP-LC-MS.

### Separation and Identification of the Extracts

On-line separation of the extracts was performed by directly combining the OSMIP-LC column with HPLC system and a QQQ mass spectrometer equipped with an ESI ionization source operated in the positive-ion scan mode (ESI-QQQ) was connected as a detector. The MIP column was protected by a prefilter (4 mm, 5 μm, from GRACE, USA). The packed OSMIP polymers were amorphic particles [30] and the background pressure of this column was very high. MeOH-acetonitrile-formate acid (75:25:0.01) was used as the mobile phase at the flow rate of 0.075 ml/min in order to avoid destroying the inner structure of column and get an ideal separation. The injected sample volume was 10 μl. Capillary potential of the QQQ MS was +4,000 V with the gas temperature at 350 °C, the gas (N_2_) flow rate at 10 L/min, the nebulizer pressure at 30 psi and the dwell time of 200 ms. Identification of the affinitive components, which had affinity with the OSMIP-LC column (the retention time was ≥ OS), was carried out based on the data in the literatures and the properties of on-line OSMIP-LC and off-line MS/MS of those components and their control samples.

### Test for the Effect on Avian Influenza Virus *in vitro*

The antivirus effect of the identified extracts on avian influenza virus H9N2 was examined *in vitro* using the established methods reported previously [32,33]. Madin-Darby canine kidney (MDCK) cells were grown in Dulbecco’s modified Eagle medium (GIBCO™, Invitrogen Corporation) supplemented with fetal bovine serum (10%). Maximum non-toxic concentration (TCD_0_) of each compound for MDCK was determined by cytopathic effect (CPE) when the cells were incubated 72 h at 37 °C with 5% CO_2_. 1 μg/milliliter TPCK-Trypsin (Sigma) was used during TCID_50_ (tissue culture infectious dose) determination. MDCK cells were incubated for 7 days and the CPE was used to determine TCID_50_ by Reed & Muench method. Influenza virus at 100 TCID_50_ were inoculated onto the MDCK cells for 1 h with occasional rocking, and then the remaining solution was discarded and solutions enriched with a testing compound was added (Each compound was tested separately). The MDCK cells incubation time was 7 - 10 days in these compounds anti-influenza virus test and terminated when the CPE of viral control was +++. Influenza virus strain A/Goose/Dalian/3/2001 (H9N2), which obtained from Animal influenza Laboratory of Ministry of Agriculture and State Key Laboratory of Veterinary Biotechnology, Harbin Veterinary Research Institute of CAAS, was used to assay each compound’s antiviral efficacy through CPE and hemagglutination inhibition (HI).

### Correlation between the Activity and Affinity

Correlation between the activity of the compounds against influenza virus H9N2 and its retention time onto the OS-MIP-LC column was analyzed using SAS 8.2 for Windows.

## Results and Discussion

### On-line Affinitive Separation of Samples with OSMIP-LC Column

In our previous work [30], OSMIP was synthesized and packed into a stainless steel LC column (150 mm × 4.6 mm i.d., 25^~^45 µm). The specific affinity tests of this OSMIP-LC column for the template molecule and other different compounds such as chlorogenic acid, phillyrin and aspirin, were performed with mass spectrometry (MS). Retention time of OS on this column was about 140 min, and the retention times of all other tested compounds were only about 35 min [30]. This indicated that the OSMIP-LC column had a very high affinity and selectivity to separate the template molecule from other compounds and the compounds without affinity and selectivity to it were not picked up. The retention times similar to or longer than the template indicates the affinity for these compounds. This column with OSMIP can, therefore, be used on-line to screen active components against influenza virus from TCH extracts.

**Figure 2:**
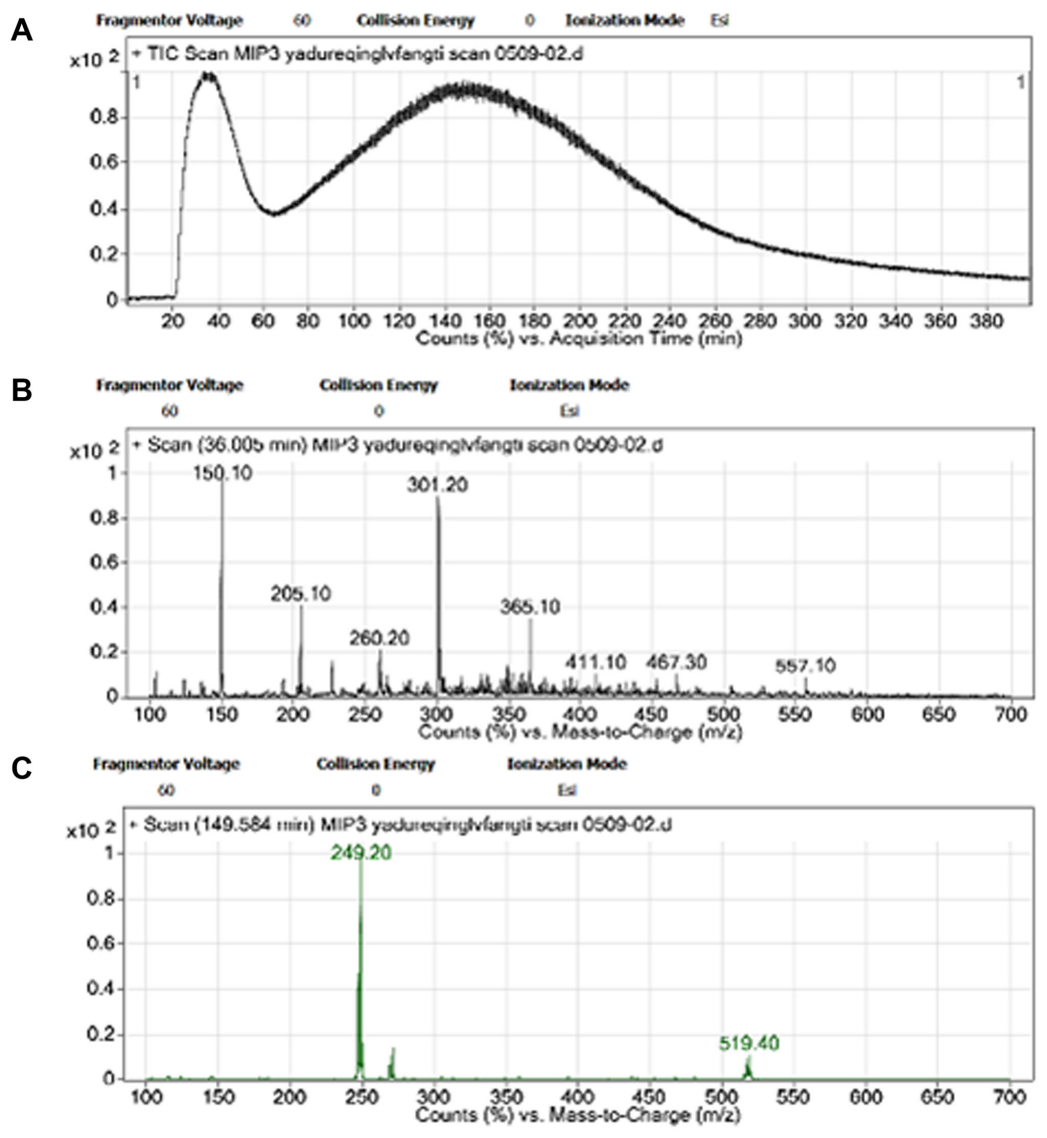
TIC of chloroform extraction from TCM preparation and MS spectra. A. TIC of chloroform extraction, B. MS spectra of the first peak, C. MS spectra of the second peak.

Several extracted samples with different polarity from different TCM were affinitively seperated by OSMIP-LC column on-line and detected by electrospray ionization (ESI) MS. Affinitive compounds were not discovered from these extracts which contained one kind of TCH. However, the affinitive component was found from chloroform extracts of TCM liquid preparation “*Yandureqing*”. The TIC and MS results are shown in Figure 2. 

### Identification of the Affinitive Component

The results of OSMIP-LC-MS affinitive separation are shown in Figure 2. There were two separated peaks in TIC (Figure 2A). Retention time of 35 min at the first peak in Figure 2 was in agreement with our previous work in which a similar first peak was observed [30]. The first peak in Figure 2B included many chemical components. Again retention time of the second peak in Figure 2, 150 min, was similar to the second peak we recorded previously [30], and it consisted of only one component. The ions *m/z* 249.20, *m/z* 271.20 and *m/z* 519.40 in Figure 2C were ions of [M + H]^+^, [M + Na]^+^ and [2M + Na]^+^ , respectively. This indicated that the molecular weight of affinitive component was about 248.20. The searching literatures of phytochemistry were focused on those TCH contained in the recipe, as this affinitive component was mainly derived from one of TCH, which was included in the recipe of TCM liquid preparation.

The studies on matrine and sophoridine were reported in several published papers [34–36]. They are stereoisomers of quinolizidine alkaloid with the molecular weights are 248.20. Their chemical structures and oxides are shown in Figure 1. The contents of these two alkaloids are much more than those of other stereoisomers in *Sophorae Flavescentis Radix* [37].

**Figure 3:**
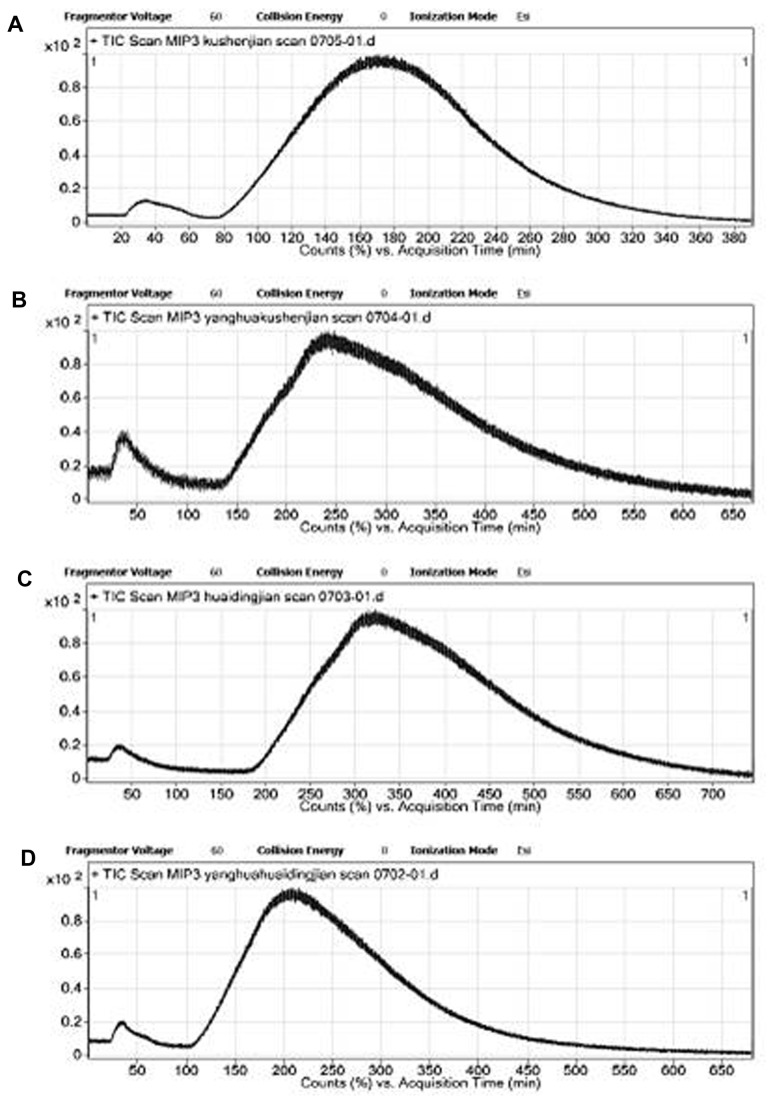
Chromatographic performance of different control compounds in OSMIP-LC-MS. A. matrine, B. oxymatrine, C. sophoridine, D. oxysophoridine.

 To identify the affinitive component, the chromatographic performance of the control compounds, including matrine, sophoridine and their oxides oxymatrine and oxysophoridine, were investigated with OSMIP-LC-MS. The chromatographic parameters are the same as the affinitive separation of OS and TCM sample. The TIC results are presented in Figure 3.

The results showed that the retention times of matrine and sophoridine in this OSMIP-LC column were approximately 170 min and 320 min, respectively (Figure 3A and C). It indicated that the retention time of the unknown affinitive component (Figure 2A, 150 min) was similar to matrine, but very different from sophoridine. Therefore, the unknown affinitive component may be matrine. Additionally, all of these four compounds had affinity in this OSMIP-LC column as their retention times were longer than OS.

**Figure 4:**
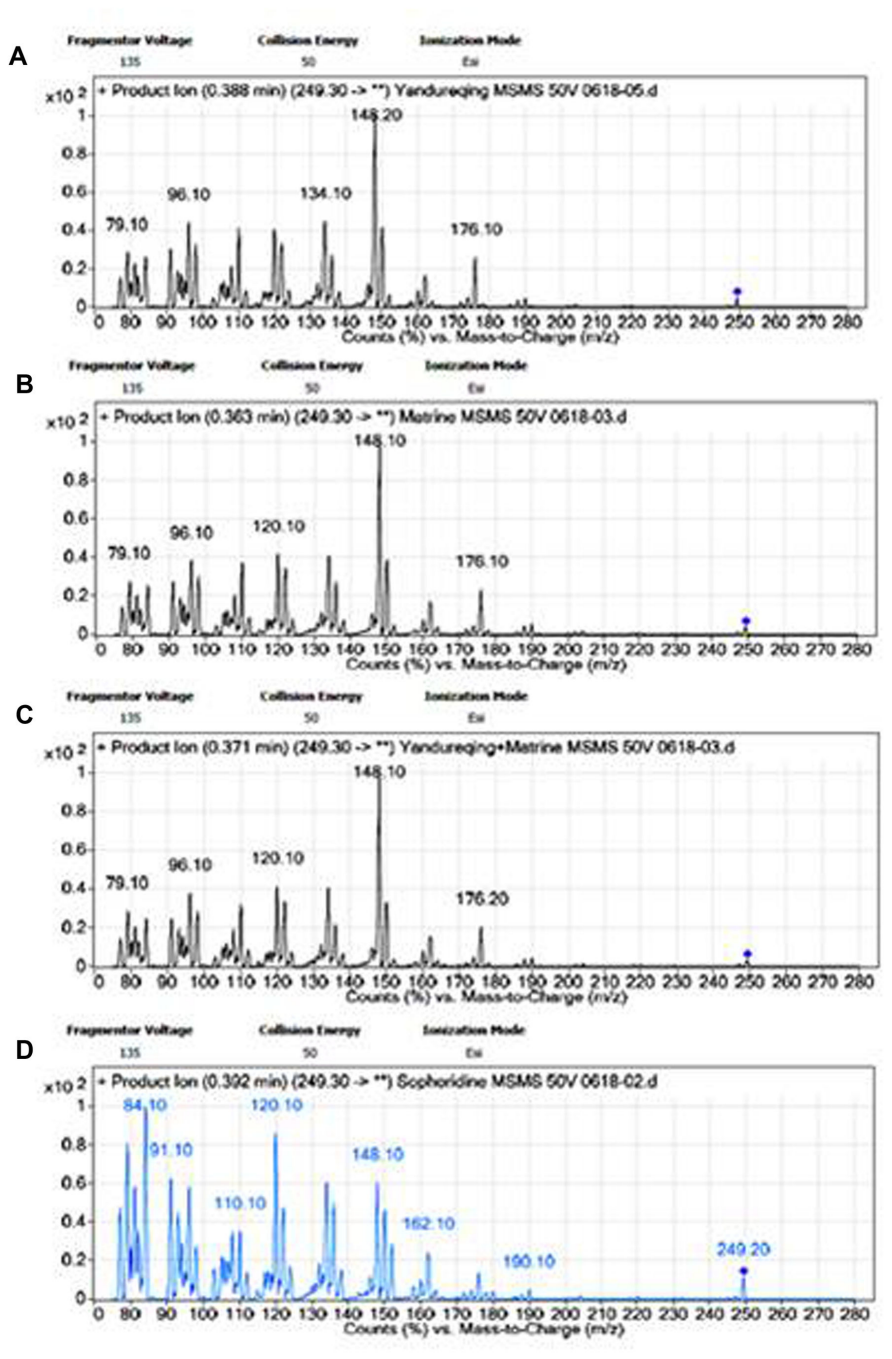
MS/MS spectra of four samples. A. chloroform extraction, B. matrine, C. chloroform extraction with matrine, D. sophoridine.

Another identification was carried out using LC-MS/MS with Agilent Zorbax SBC_18_ (30 mm×2.1 mm, 3.5 μm) column protected by a prefilter (4 mm, 5 μm, from GRACE, USA) at a flow rate of 400 μl/min. The liquid phase consisted of MeOH-water-5 M ammonium formate (75:25:0.1). An Agilent 6410A QQQ MS with ESI source interface operated in the positive-ion scan mode was employed for MS/MS analysis. The precursor ion *m/z* was 249.30, and fragmentor voltage and collision energy were 135 V and 50 eV, respectively. The injection volume of each sample was 5 μl. And other LC and MS parameters were as the same as the above affinitive separation. Four samples of MeOH solution were investigated with LC-MS/MS. These samples contained control compounds matrine and sophoridine, chloroform extraction of TCM liquid preparation and the chloroform extraction with control compound matrine. The results of MS/MS spectra are shown in Figure 4.

The results of MS/MS spectra showed that the three samples of chloroform extraction, matrine and chloroform extraction with matrine had almost identical MS/MS spectra under the same LC-MS/MS parameters, and their characterized product ions were *m*/*z* 249 → 148 and 150. However, the spectrum (abundance) of sophoridine was different from those (Figure 4D). The characteristic fragmentation behavior of those four samples indicated the affinitve component was matrine.

 According to the results of chromatography (retention time) in OSMIP-LC column and product ions of *m/z* 249, the affinitive component was identified as matrine.

### Bioactivity of the Compound against Avian Influenza Virus *in vitro*

**Figure 5:**
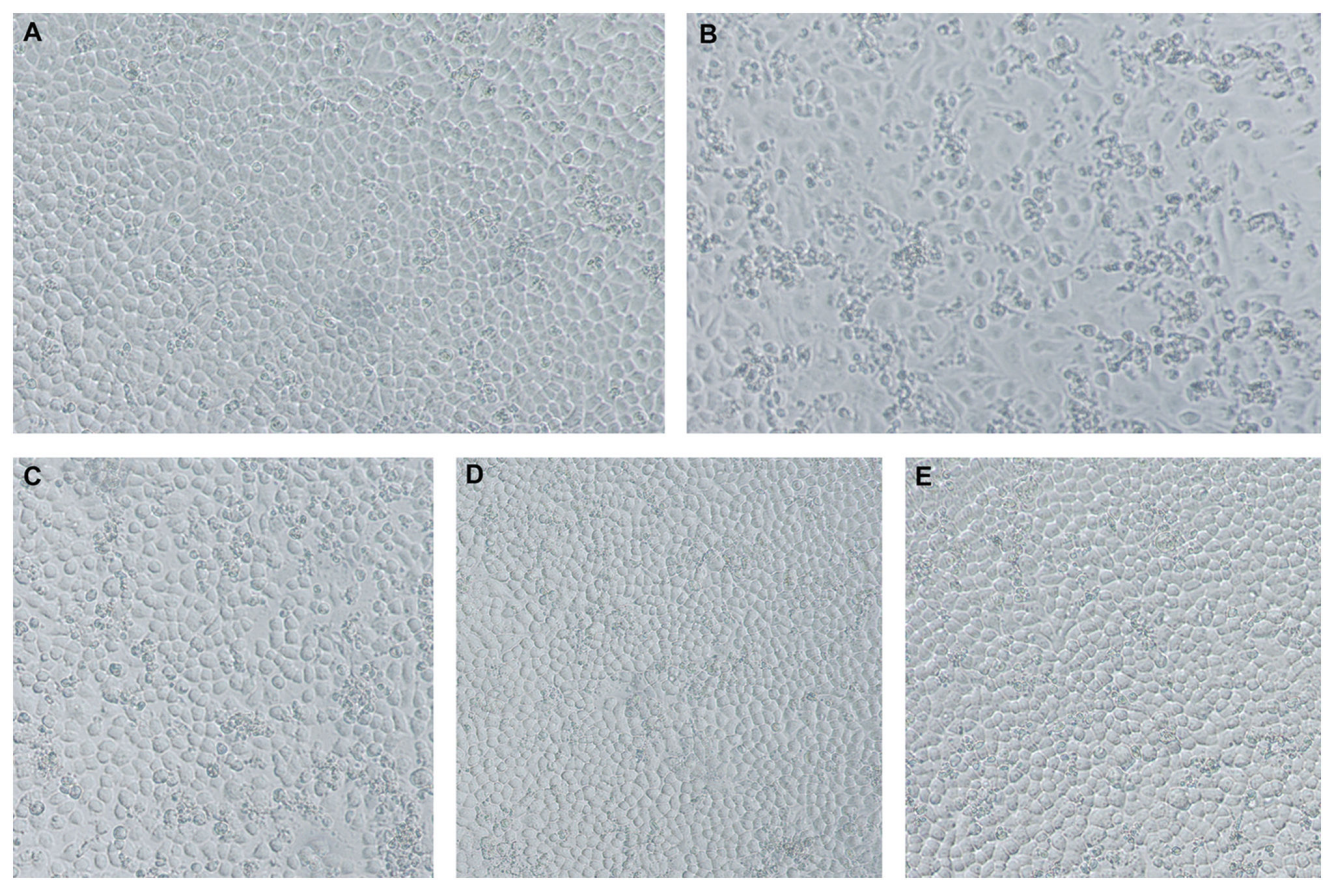
Photos of matrine against H9N2 *in vitro* when cytopathic of viral control was +++. A. Blank control, B. Viral control, C. 0.4 mg/ml matrine, D. 0.2 mg/ml matrine, E. 0.1 mg/ml matrine.

The results of bioactivity tests are shown in Table 1 and Figure 5. 

**Table 1:** Activity of compounds against H9N2 *in* *vitro*.

Compound	TCD_0_	Activity		
		CPE		HI
OS (template)	0.5mg/ml	The CPE of 0.5mg/ml OS-treated group was more severe than viral control group within 48 h. The CPE of groups 0.25, 0.125 and 0.0625 mg/ml were milder than viral control at 72 h.		No hemagglutination (H) was found in all tested groups at 24, 48 or 72 h.
aspirin	>0.5mg/ml	No differences between all treatment and control groups.		
chlorogenic acid	16 μg/ml	No differences between all treatment and control groups.		
phillyrin	1 mg/ml	The CPE of 0.5 and 0.25 mg/ml phillyrin-treated groups were more severe than that of the viral control group within 48 h.		There was no H in 0.25 mg/ml phillyrin-treated group at 24 h and in 0.5mg/ml phillyrin-treated group at 24 or 48 h.
matrine	0.4 mg/ml	The CPE of 0.4, 0.2 and 0.1 mg/ml matrine groups were milder than that of the viral control group.		There was no H in 0.2 mg/ml matrine-treated group at 24 h and in 0.4mg/ml matrine-treated group at 24 or 48 h.
oxymatrine	>2 mg/ml	No differences between all treatment and control groups.		
sophoridine	>2 mg/ml	The CPE of 2 mg/ml sophoridine-treated group was more severe than that of the viral control at 48 h. The CPE of other test groups with low concentration were consistent with that of the viral control at 48 h.		There was no H of 2 mg/ml sophoridine-treated group at any test time points. But the rest test groups had H at all test time points.
oxysophoridine	>2 mg/ml	No differences between all treatment and control groups		

The results in Table 1 showed that both OS and matrine had activity of alleviating CPE and HI against H9N2 *in vitro*. The results in Figure 5 showed that matrine could protect MDCK cells from H9N2 in a dose-dependent manner. Sophoridine, as stereoisomer of matrine, had HI activity only at concentration of TCD_0_. Phyllirin had HI activity whilst it could not alleviate CPE. All of other tested compounds didn’t produce any activities against H9N2 *in vitro* in this study.

### Correlation Analysis between bioactivity and Affinity

The retention times of matrine, oxymatrine, sophoridine and oxysophoridine were similar to or longer than the OS, so these four compounds had affinity with the OSMIP-LC column. Matrine had activities both of alleviating CPE and HI while sophoridine reduced HI only at the highest tested concentration. The other three tested compounds aspirin, chlorogenic acid and phillyrin did not affiliate to the prepared column. But phillyrin could still have HI. Therefore qualitative and quantitative correlate analysis between activity and affinity was required. 

The compound with or without affinity to the prepared column was defined by “1” or “0” respectively and that with or without bioactivity against H9N2 *in vitro* (alleviating CPE and/or HI) was defined by “1” or “0”. The results of affinity and activity were described in Table 2.

**Table 2:** Retention time, affinity and activity of each tested compounds.

Compounds	Retention time	Affinity	Activity (see Table 1)	
	(approximately)		qualitative	semi-quantitative
OS (template)	140 min [30]	1	1	3
aspirin	35 min [30]	0	0	0
chlorogenic acid	35 min [30]	0	0	0
phillyrin	35 min [30]	0	1	1
matrine	150 min (Figure 2A) 170 min (Figure 3A)	1	1	3
oxymatrine	240 min (Figure 3B)	1	0	0
sophoridine	320 min (Figure 3C)	1	1	1
oxysophoridine	210 min (Figure 3D)	1	0	0

The statistical analysis showed a P of 0.462, and correlation coefficient *R* between affinity and activity of 0.500. These indicated no statistically significant correlation between the tested compounds - OSMIP-LC column affinity and the qualitative activity against H9N2 *in vitro*.

Results of the bioactivity showed that matrine had similar activities with the template OS, and its activity was higher than all other tested compounds. In addition, affinitive separation and identification results showed that the retention time of matrine was closest to that of the OS in all tested compounds. The three compounds (aspirin, chlorogenic acid phillyrin) hardly have affinity with the prepared column and activity. On the other hand, the other three tested compounds with more affinity (longer retention times) to the column hardly show any activity. 

**Figure 6:**
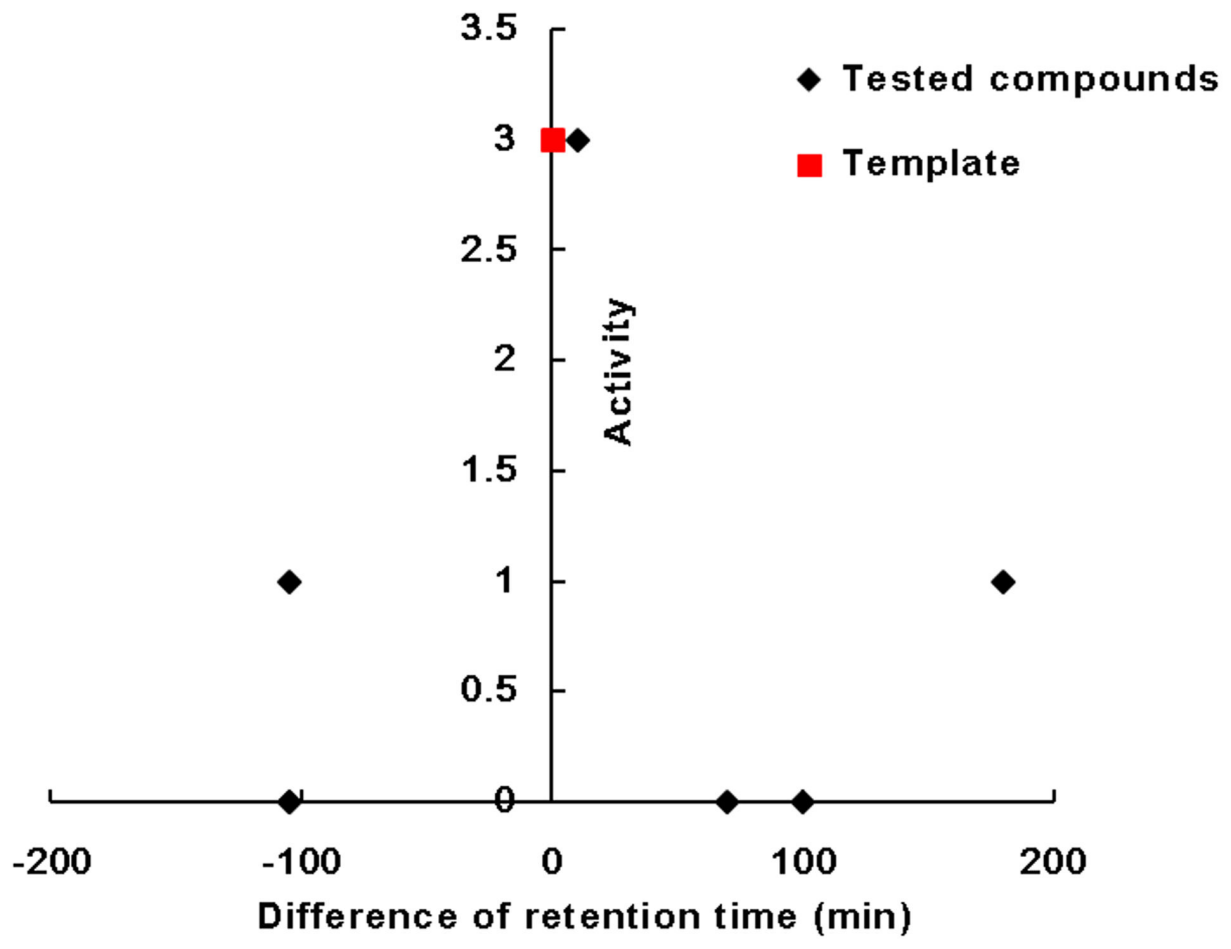
Trend graph between activity and retention time difference.

Semi-quantitative data were used to define different categories of activity, in which “0” presented non-activity, “1” meant little activity (phillyrin and sophoridine) and “3” was the highest activity (OS and matrine). The semi-quantitative data of their activity are shown in the last column of Table 2. The trend graph between difference of retention times of the tested compounds from the template and the activity is shown in Figure 6.

The trend graph indicated that less difference of retention time might confer higher activity.

(E)-piceatannol is a natural potential anti-epidermal growth factor receptor (EGFR) inhibitor. The (*E*)-piceatannol MIP was prepared by Xu et al [16]. Two different compounds butein and quercetin with potent anti-ENGR tyrosine kinase activity and the template itself were recognized by the polymer from the crude extracts of a Chinese traditional Tibetan medicinal herb *Caragana jubata*. Affinity and selectivity for these inhibitors and another three tested compounds combined with the template in this herb were evaluated in the chromatographic mode. The results of Xu et al. [16] showed that the chromatographic behavior of the analytes was consistent with their activity values: the more active inhibitor, the longer on the MIP.

The results of our work showed that chromatographic behavior of the analytes had some correlation with their bioactivities. However, it was different from the conclusion by Xu et al [16]. We, therefore, raised the hypothesis that the less difference of chromatographic behavior between the analytes and the template, the more similar bioactivities. 

### Comparison of Chemical structure between template and affinitive component(s)

Most of the published papers about MIP mainly focused on the template molecule itself, the chiral isomer of the template, or differences of the congener compounds between several groups and the template (see Table S1), whereas their chemical structures of oseltamivir and matrine are very different (see Figure 1). From a structure-activity relationship (SAR) point of view, most inhibitors against the same receptor often adopt a similar or even common binding model and thus tend to possess similar structures in terms of size, shape, and functional groups. Therefore, the structure of compound with affinity to the prepared OSMIP-LC column should show similarity to the template OS. However, matrine, oxymatrine, sophoridine and oxysophoridine are quinolizidine alkaloids, and their two-dimensional structures are different from the OS (see Figure 1). 

**Figure 7:**
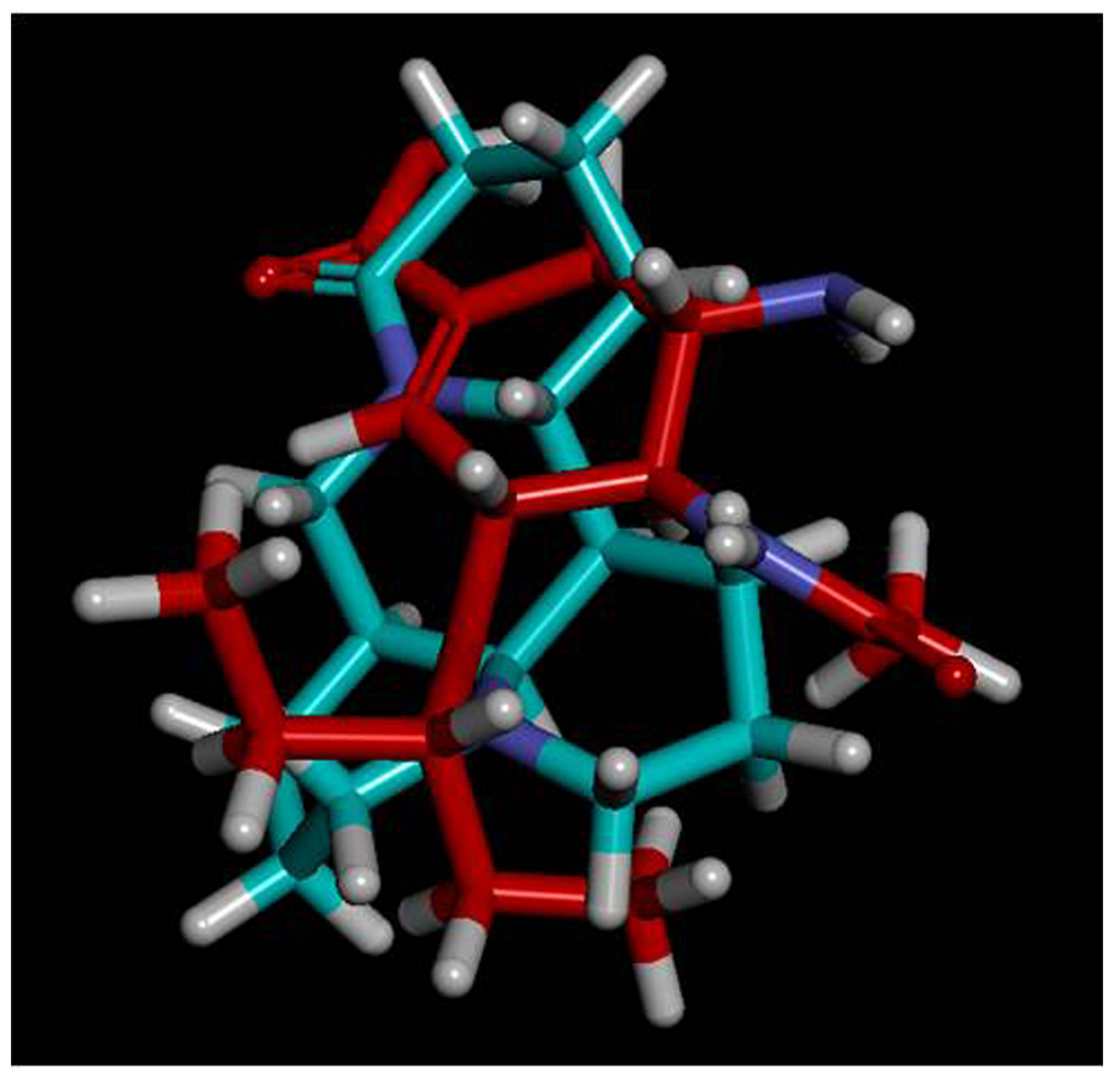
Stereostructures overlay of OS and matrine. (The OS was labeled as red and matrine as green).

The preferred conformations of oseltamivir carboxylate (a hydrolysate of OS *in vivo* with direct action) and matrine were compared when they possess minimum energy as calculated with Discovery Studio 3.1.1 (Accelrys, USA). The finding showed the similarity of their preferred conformations (see Figure 7). 

**Figure 8:**
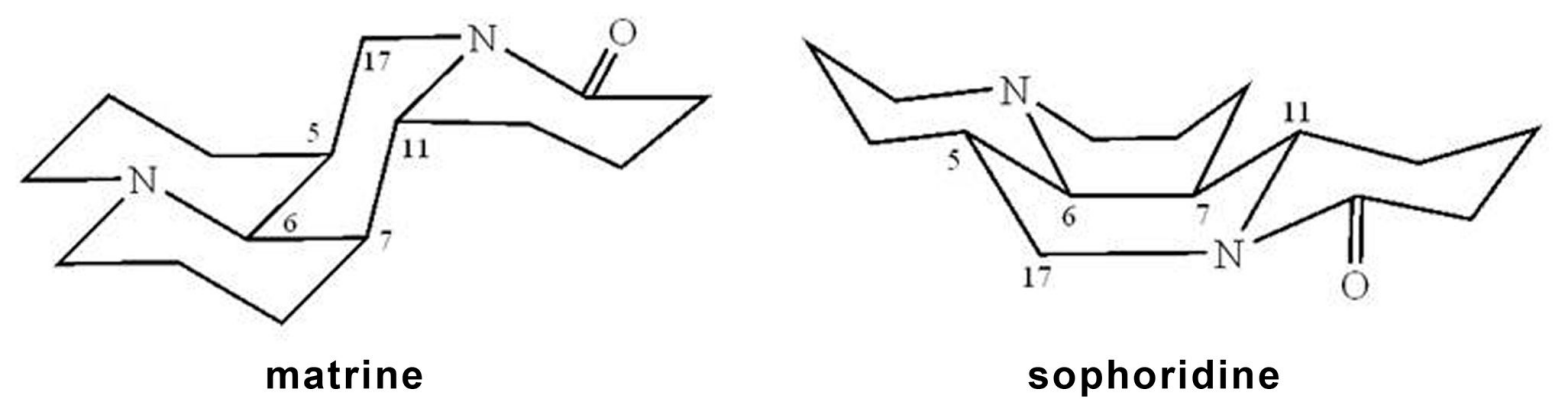
Stereostructures of matrine and sophoridine [34].

Matrine and sophoridine had high and low bioactivities against H9N2 *in vitro*, respectively, but the oxides oxymatrine and oxysophoridine did not show any similar bioactivities in our present study. Therefore, the site N (1) of these compounds might be one of the key sites for their bioactivities against H9N2 *in vitro*. Although the two-dimensional structure of matrine and sophoridine are very similar except their difference in C (5), their bioactivities were very different. Pu et al. [34] had proved that their stereostructures are different and those are shown in Figure 8. Therefore, their bioactivity difference against H9N2 could be explained by their stereostructures.

### Pharmacological activities and toxicity of matrine

Matrine is one of effective component of commonly used TCM of *Sophorae Flavescentis Radix*, *Sophorae Tonkinensis Radix et Rhizoma*, and plant of *Sophora alopecuroides* L.. It has reported that matrine has many pharmacological activities, such as antifibrotic effects for liver, antiarrhythmic, anti-inflammatory, anti-tumor, and so on.

 Matrine also has good antiviral activities for hepatitis virus, Coxsackie virus, herpes simplex virus and so on. Yang et al. [38] evaluated antiviral effect of matrine against enterovirus 71 *in vitro* and *in vivo*. Matrine could suppress the viral RNA copy number on rhabdomyosarcoma cells. Moreover, matrine treatment of mice challenged with a lethal dose of enterovirus 71 reduced the mortality and relieved clinical symptoms. The results showed that matrine may represent a potential therapeutic agent for enterovirus 71 infection.

As far as we know, there was rarely paper reported that matrine had bioactivity against influenza virus *in vitro* or *in vivo*. The paper [29] showed pterocarpans and flavanones derived from *Sophora flavescens* display neuraminidase inhibition, but not quinolizidine alkaloid matrine.

Wang et al. [39] had investigated matrine’s toxicity in mice. The acute toxicity test of matrine indicated that the tolerable dose of matrine is above 80 mg/kg in *Kunming* mice, and the LD_50_ was 157.13 mg/kg (95% confidence limit, 88.08-280.31 mg/kg). Morphological observation revealed degenerative changes of the nerve cells in the brain tissue of the mice.

## Conclusions

MIP has two important characters: steric memory (size and shape) and chemical memory (spatial arrangement of the complementary functionality). They are used for separating template or its analogues from the matrix, trapping different types of active compounds from the herbs or fermentation broth and recognizing the inhibitors according to their bioactivities. Our results showed that the application of imprinting polymers as surrogates for native receptors in drug discovery is practicable. 

The prepared OS-MIPs were synthesized by non-covalent interactions, the possible interactions between template OS and MIP were weak interactions. If the interactions between analytes and OS-MIP were much stronger, it will produce too much longer retention time in this study and they might not have similar bioactiveties as their interactions were different from that of template. It is proposed that bioactivies of affinitive compounds were correlated with the difference of their retention time from template and less difference may illustrate more similar bioactivities with the template.

To date, molecularly imprinted materials as novel sorbent with predetermined selectivity have been used in a number of applications. Most of the studies mainly focus on the template molecule itself, the chiral isomer of the template, or the congener compounds different in several groups from the template [17,40–43], whereas using MIP to extract different types of compounds with similar bioactivities to the template from herbs [16,17,40], especially from the compound TCM preparation has rarely been reported. This research is the first attempt to use a molecular imprinted polymer with an active influenza virus neuraminidase inhibitor oseltamivir to simulate the receptor in recognizing different inhibitors according to their bioactivities. 

Looking at MIPs as artificial receptors (antibody-mimics), one can imagine that a polymer imprinted with a known drug to screen a combinatorial or natural library for alternative substances (agonists or antagonists), especially when the biological receptor itself is not readily available. Therefore, molecular imprinting technology as non-biological method has the potential to play an important role in drug development. 

Matrine as a quinolizidine alkaloid has many pharmacological actions with milder toxicity [39]. The present work was the first time to report its bioactivity against H9N2 influenza virus. Matrine may be a potential candidate to be developed as a novel drug against influenza.

## Supporting Information

Table S1 **Chemical structures comparison of template and affinitive component(s).**
(DOC)
