# Supplementary material for: A Non-Biological Method for Screening Active Components against Influenza Virus from Traditional Chinese Medicine by Coupling a LC Column with Oseltamivir Molecularly Imprinted Polymers
Source: PLoS One. 2013 Dec 26;8(12):e84458. doi: 10.1371/journal.pone.0084458 (PMC3873415; doi:10.1371/journal.pone.0084458)
Supplement: Table S1 — Chemical structures comparison of template and affinitive component(s). (DOC) [file pone.0084458.s001.doc]

**Supporting Information**

**A Non-Biological Method for Screening Active Components against Influenza Virus from Traditional Chinese Medicine by Coupling a LC Column with Oseltamivir Molecularly Imprinted Polymers**

Ya-Jun Yang, Jian-Yong Li*, Xi-Wang Liu, Ji-Yu Zhang, Yu-Rong Liu, Bing Li

* Correspondence to: lijy1971@163.com

**Comparison of Chemical structure between template and affinitive component(s).** Most of the published papers about MIP mainly focused on the template molecule itself, the chiral isomer of the template, or differences of the congener compounds between several groups and the template. The chemical steuctures of those templates and affinitive compounds are shown in Table S1.

**Table S1** Chemical structures comparison of template and affinitive component(s)

| No. | Template | Affinitive Component(s) | | Literature(s) |
| --- | --- | --- | --- | --- |
| 1 |  |  | | This work. |
|  | oseltamivir | matrine | |  |
| 2 |  |  | | [1] |
|  | propyl gallate | protocatechuic acid | |  |
| 3 |  |  |  | [2] |
|  | (*E*)-piceatannol | quercetin | butein |  |
| 4 |  |  |  | [3] |
|  | harman | harmine | harmaline |  |
| 5 |  |  |  | [4, 5] |
|  | quercetin | isorhamnetin [4] | kaempferol [5] |  |

**Reference**

1. Huang M, Pang W, Zhang J, Lin S, Hu J (2012) A target analogue imprinted polymer for the recognition of antiplatelet active ingredients in *Radix Salviae Miltiorrhizae* by LC/MS/MS. J Pharm Biomed Aanl 58:12-18.

2. Zhu L, Chen L, Xu X (2003) Application of a Molecularly Imprinted Polymer for the Effective Recognition of Different Anti-Epidermal Growth Factor Receptor Inhibitors*.* Anal Chem 75: 6381-6387.

3. Xie J, Zhu L, Xu X (2002) Affinitive Separation and On-Line Identification of Antitumor Components from *Peganum nigellastrum* by Coupling a Chromatographic Column of Target Analogue Imprinted Polymer with Mass Spectrometry. Anal Chem 74: 2352-2360.

4. Zhou L, Xie JC, Ge YF, Xu XJ (2002) The Application of Molecular Imprinting Technology for *Hippophae rhamnoidse* Linn. Function Integrants Extraction. Acta Phys - Chim Sin 18: 808-811.

5. Xie JC, Luo HP, Zhu LL, Zhou L, Li CX, et al. (2001) Extracting Active Compounds from Herbs Using Molecular Imprinting Technology. Acta Phys - Chim Sin 17: 582-585.
